# Identification of Brimonidine as a Novel Substrate of Organic Cation Transporters OCT2 and MATE1 Expressed in Human Eye

**DOI:** 10.1167/iovs.67.4.4

**Published:** 2026-04-02

**Authors:** Charlotte Kölz, Claudia Neul, Ute Hofmann, Julia C. Dressler, Daniela Süsskind, Bernd Wissinger, Matthias Schwab, Anne T. Nies

**Affiliations:** 1 Dr. Margarete Fischer-Bosch Institute of Clinical Pharmacology, Stuttgart, and University of Tübingen, Tübingen, Germany; 2 Department of Clinical Pharmacology, University of Tübingen, Tübingen, Germany; 3 Institute for Ophthalmic Research, Center for Ophthalmology, University of Tübingen, Tübingen, Germany; 4 Department of Pharmacy and Biochemistry, University of Tübingen, Tübingen, Germany

**Keywords:** brimonidine, membrane transporters, solute carrier transporters, SLC47A1, SLC22A2

## Abstract

**Purpose:**

Brimonidine, a selective α_2_-adrenergic agonist, is widely used in topical therapy for ocular hypertension or primary open-angle glaucoma. However, effective delivery is hindered by multiple ocular surface barriers. Because brimonidine is a cationic drug, we hypothesized that organic cation transporters (OCTs) and multidrug and toxin extrusion proteins (MATEs) are involved in brimonidine uptake in the human eye. This study aimed to determine if brimonidine is transported by OCT1, OCT2, OCT3, MATE1, and MATE2K and if the identified transporters are localized in anterior eye structures.

**Methods:**

Uptake studies were performed using HEK293 cells stably expressing OCT1, OCT2, OCT3, MATE1, and MATE2K. Intracellular brimonidine accumulation was analyzed by mass spectrometry. Immunohistochemistry of glaucomatous human eyes was used to localize relevant transporters in anterior ocular structures.

**Results:**

Brimonidine was transported by OCT2 and MATE1 but not by OCT1, OCT3, or MATE2K. Uptake was time- and concentration-dependent. Both OCT2 and MATE1 were expressed in the cornea, the conjunctiva, and the ciliary body.

**Conclusions:**

These results imply that OCT2 and MATE1 may play a role in brimonidine uptake into the human eye and may contribute to the interindividual variability of brimonidine concentrations and effects.

Glaucoma is a group of progressive eye diseases that damage the optic nerve and remains one of the leading causes of blindness worldwide.^1^^–^^3^ Primary open-angle glaucoma (POAG) is the most common form, accounting for approximately 75% of all cases.^1^ A recent meta-analysis of 50 studies estimated a substantial global population of more than 68 million adults older than 40 years with POAG.^3^ Projections suggest that this number will increase to nearly 80 million cases by 2040.^2^ Since visual loss from glaucoma is irreversible, it is essential to identify risk factors and effective treatments. Elevated intraocular pressure (IOP) has been identified as a major risk factor for optic nerve damage,^4^^–^^6^ and lowering IOP remains the cornerstone of therapy.

Topical ocular hypotensive medications are the primary means of reducing IOP. Large landmark randomized controlled trials have demonstrated that these agents can delay or prevent glaucoma, establishing them as the preferred approach to glaucoma treatment.^4^^,^^6^ Currently, there are six major classes of IOP-lowering drugs: prostaglandin analogues, β-adrenergic receptor antagonists (β-blockers), α_2_-selective adrenergic agonists, carbonic anhydrase inhibitors, Rho kinase inhibitors, and parasympathomimetics, each class with a different mechanism of action, side effect profile, and efficacy.^4^^,^^6^^–^^8^

Among these agents, the α_2_-selective adrenergic agonist brimonidine occupies a unique therapeutic niche due to its dual mechanism of action.^4^^,^^6^^,^^9^ First, it reduces aqueous humor production by inhibiting adenylate cyclase in the ciliary epithelium. Second, it increases aqueous humor outflow through the uveoscleral and trabecular pathways. This combination distinguishes brimonidine from other drug classes. Although prostaglandin analogues or β-blockers are often preferred as initial therapy, brimonidine is frequently prescribed when these agents are contraindicated. For instance, β-blockers may be unsuitable for patients with asthma, bradycardia, chronic obstructive pulmonary disease, or a history of cardiac failure. On the other hand, prostaglandins are sometimes poorly tolerated due to ocular surface disease.^5^^–^^8^ In these cases, brimonidine is an important alternative therapy.

However, effective ocular delivery of brimonidine remains challenging. After topical application, drugs must cross several ocular barriers, such as the cornea, conjunctiva, and ciliary body, to reach their intraocular targets.^10^^,^^11^ These barriers contribute to extremely low ocular bioavailability; typically, less than 5% of the topically administered dose reaches the aqueous humor.^12^ Furthermore, clinical studies have reported marked interindividual variability in intraocular drug concentrations. For example, such variability has been described for the prostaglandin analogue latanoprost, where aqueous humor concentrations varied considerably between patients despite similar dosing regimens.^13^^,^^14^

Emerging evidence suggests that membrane transporters may contribute to this variability.^15^ In systemic pharmacology, drug transporters are widely recognized as key determinants of tissue distribution and drug exposure (reviewed, e.g., in various studies^16^^–^^20^). For instance, organic anion transporting polypeptides (OATPs), organic cation transporters (OCTs), and multidrug and toxin extrusion proteins (MATEs) play major roles in drug handling by the liver and kidney. In the field of ophthalmology, however, much less information on transporters is available. Thus far, only one transporter has been implicated in the intraocular delivery of latanoprost: the organic anion transporter OATP2A1.^13^ Genetic variation in the *SLCO2A1* gene, which encodes OATP2A1, has been associated with a reduced IOP-lowering effect in Han Chinese patients with POAG or ocular hypertension.^21^ This provides clinical evidence that genetic variants in drug transporters may affect the efficacy of IOP-lowering drugs.

Brimonidine, like many systemically important drugs, is a basic compound with a positive charge at physiological pH (Fig. 1A). These physicochemical properties make it a potential substrate for OCTs and MATEs. These transporters mediate the transport of a wide range of cationic compounds and clinically relevant drugs such as metformin, oxaliplatin, and tramadol (reviewed, e.g., in various studies^16^^–^^20^). Importantly, common genetic variants in OCT1 and OCT2 can alter transporter function, thereby affecting systemic drug disposition and therapeutic response.^16^^,^^17^^,^^19^ Despite their well-established roles in hepatic and renal pharmacology, the involvement of OCTs and MATEs in ocular drug delivery has not been investigated.

**Figure 1.**
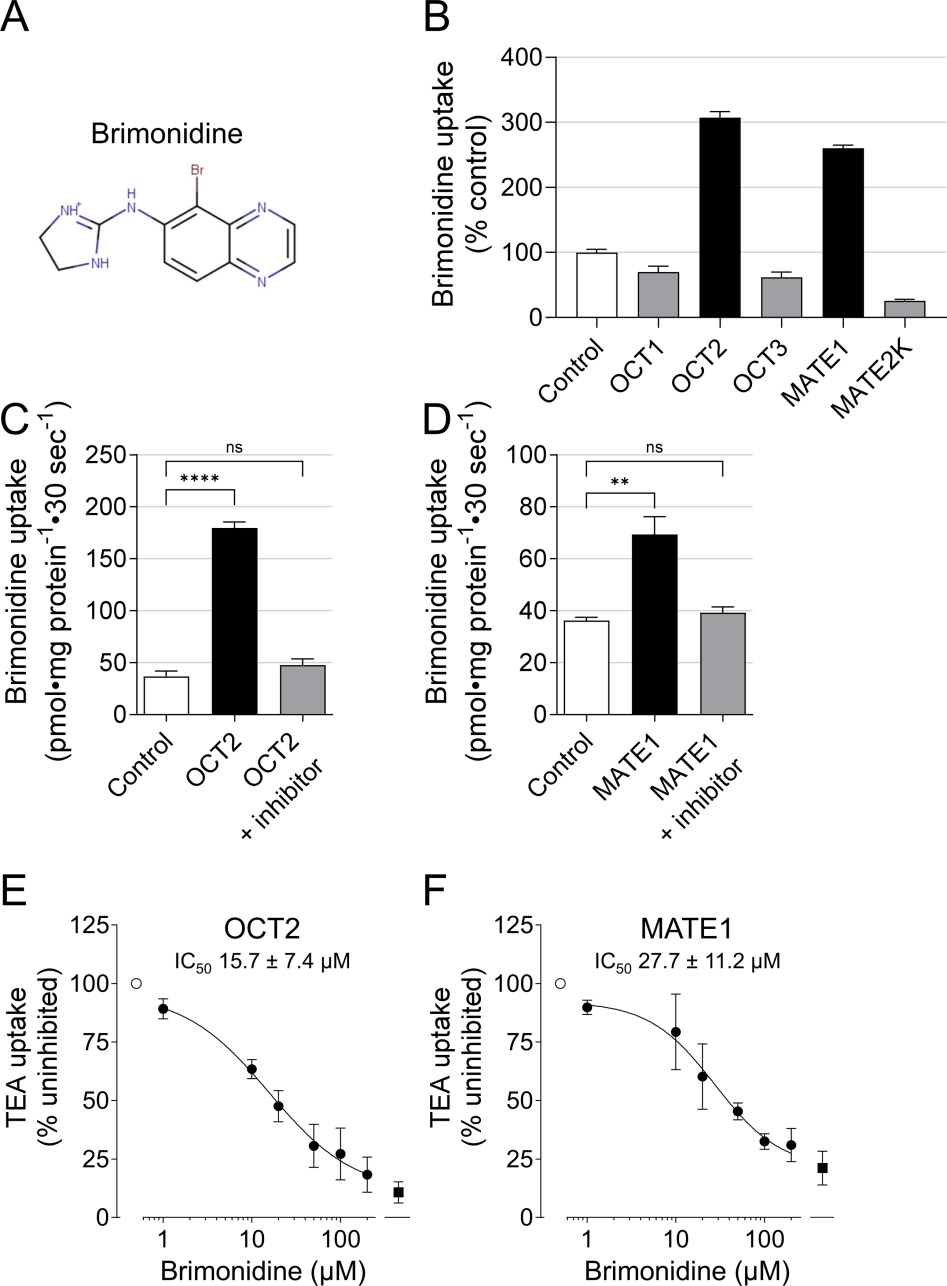
Identification of OCT2 and MATE1 as brimonidine transporters. (**A**) Structure of brimonidine at a pH of 7.4. (**B**) Screening of brimonidine transport by OCT1, OCT2, OCT3, MATE1, and MATE2K. Brimonidine uptake (5 µM) was measured after a 10-minute incubation and compared to uptake into vector-transfected cells (control = 100%). Data are mean ± SD from three wells from one experiment. (**C**, **D**) Inhibition of OCT2- and MATE1-mediated brimonidine uptake by the organic cation transport inhibitor MPP. Brimonidine uptake (5 µM) into OCT2- or MATE1-expressing cells or into vector-transfected control cells was measured after a 30-second incubation in the presence or absence of 2 mM MPP. Data are mean ± SEM of three independent experiments, each performed in triplicate. (**E**, **F**) Inhibition of the uptake of the prototypic substrate TEA into OCT2- and MATE1-expressing cells by brimonidine. Transporter-expressing cells were incubated with 100 µM TEA in the presence of different brimonidine concentrations, and cellular uptake was determined after 30 seconds (OCT2) or 2 minutes (MATE1) (*filled circles*). Data are presented as the percentage of uninhibited TEA uptake in the absence of brimonidine (100%, *open circles*). The *filled square* indicates TEA accumulation in the presence of the organic cation inhibitor MPP (2 mM) used as a positive control inhibitor. Data are mean ± SEM of three independent experiments, each performed in triplicate. **P < 0.01; ****P < 0.0001; ns, not significant.

Given the variability in intraocular brimonidine concentrations observed in patients,^22^ transporter-mediated uptake is a compelling yet unexplored mechanism. Determining whether brimonidine is a substrate of OCTs or MATEs and identifying the relevant transporters expressed in ocular tissues could provide new insights into variability in clinical response and highlight novel targets for drug delivery.

Therefore, the objectives of the present study were threefold: (1) to establish a mechanistic basis for OCT- and MATE-mediated brimonidine transport using well-described cell systems, (2) to evaluate the effect of common genetic variants in the identified transporters on their activity in vitro, and (3) to localize the identified transporters in anterior eye structures using human tissue. Our findings identify OCT2 and MATE1 as brimonidine transporters in the cornea, conjunctiva, and ciliary body. These results support the role of these transporters in intraocular delivery and suggest a mechanistic basis for intraindividual variability in intraocular brimonidine concentrations.

## Material and Methods

### Chemicals

Brimonidine tartrate (#SC-217788) and the internal standard brimonidine-d4 D-tartrate (#SC-217789) were purchased from Santa Cruz Biotechnology, Inc. (Heidelberg, Germany). [1-^14^C]Tetraethylammonium bromide ([^14^C]TEA, 130 MBq/mmol) was from PerkinElmer LAS (Germany) GmbH (Rodgau-Juegesheim, Germany) (NEC-298). All other chemicals were of analytical grade and obtained from Sigma-Aldrich/Merck (Darmstadt, Germany) if not indicated otherwise.

### Cell Lines Overexpressing OCT1, OCT2, OCT3, MATE1, and MATE2K

Human embryonic kidney (HEK) 293 cells stably transfected with *SLC22A1*, *SLC22A2*, *SLC22A3*, *SLC47A1*, and *SLC47A2* cDNA encoding for human OCT1, OCT2, OCT3, MATE1, and MATE2K membrane transporter proteins, respectively, were generated and cultivated as described.^23^ As previously demonstrated,^23^^–^^25^ the recombinant expressed transporter proteins were integrated into the membrane and functionally active using prototypical substrates and inhibitors. For the uptake experiments, cells were seeded in poly-L-lysine–coated 24-well plates (Thomas Geyer GmbH & Co. KG, Renningen, Germany) at a density of 5 × 10^5^ cells/well and grown to confluence for 2 days. After 24 hours, 5 mM sodium butyrate was added to the OCT- and MATE1-expressing cells in order to induce the expression of recombinant transporters.

### Uptake Studies With Brimonidine

Uptake studies with the stably transfected HEK cells and the respective vector control cells were performed as described.^23^ Cells were incubated with uptake buffer containing brimonidine with the indicated concentrations and for the indicated time periods. For the inhibition studies, the prototypical organic cation and inhibitor 1-methyl-4-phenylpyridinium (MPP)^17^ was used at a final concentration of 2 mM. At the beginning of the experiments, cells were washed with prewarmed uptake buffer (37°C) and incubated in the buffer for 10 minutes at 37°C and 5% CO_2_. Subsequently, cells were incubated with the brimonidine solutions in the absence or presence of MPP for the indicated time periods. Cells were washed three times with ice-cold uptake buffer, followed by two washing steps with ice-cold PBS. Afterward, cells were lysed in acetonitrile/H_2_O 1:1 (v/v) containing 0.1% formic acid and the internal standard brimonidine-d4 (5 µM). The intracellular accumulation of brimonidine was measured using liquid chromatography/tandem mass spectrometry (LC/MS/MS). The amount of brimonidine was related to the total amount of cellular proteins, which was measured by the method by Smith et al.^26^

### Quantification of Brimonidine by LC/MS/MS

LC-MS analysis was performed on an Agilent 6120 MSD (Agilent, Waldbronn, Germany) coupled to an HPLC 1200 system (Agilent) consisting of binary pump G1312B, degasser G1379B, autosampler G1329B, and column thermostat G1316A. Ionization was achieved using electrospray ionization in positive polarity, with nitrogen employed as both the drying and nebulizer gas.

HPLC separation was achieved at 30°C on an Accucore AQ C18 column (50 × 2.1 mm ID, 2.6 µm particle size; Thermo Fisher Scientific, Dreieich, Germany) using (A) 0.1% formic acid in water and (B) 0.1% formic acid in acetonitrile as mobile phases at a flow rate of 0.23 mL·min^−1^. Gradient runs were programmed as follows: 7% B from 0 to 1 minute, increase to 60% B to 6 minutes, 60% B to 6.5 minutes, and then reequilibration. The mass spectrometer was operated in the selected ion monitoring mode using *m/z* 292 for brimonidine, *m/z* 298 for brimonidine-d_4_, and a fragmentor of 140. The following MS parameters were applied: nebulizer 50 psi, dry gas 10 L/min, dry temperature 350°C, and capillary 4000 V.

Calibration samples were prepared in uptake buffer/acetonitrile 1:1 (v/v) containing 1% formic acid and 1 µM internal standard brimonidine-d_4_, in the concentration range from 6.25 to 1000 nM for brimonidine. Calibration samples were processed as the samples and analyzed together with the unknown samples. Calibration curves based on internal standard calibration were obtained by weighted (1/x) linear regression for the peak area ratio of the analyte to the internal standard against the amount of the analyte. The concentration in unknown samples was obtained from the regression line. Assay accuracy and precision were determined by analyzing quality controls that were prepared like the calibration samples.

### Uptake Studies With Prototypical Substrate TEA

Uptake studies with the organic cation and prototypical substrate TEA were carried out using 100 µM [^14^C]TEA as described.^23^ For the determination of IC_50_ values of brimonidine for the inhibition of TEA uptake, six different brimonidine concentrations between 1 and 200 µM were used. The prototypical organic cation MPP was used as a positive control inhibitor at a concentration of 2 mM.

### Data Analysis

**Figure 2.**
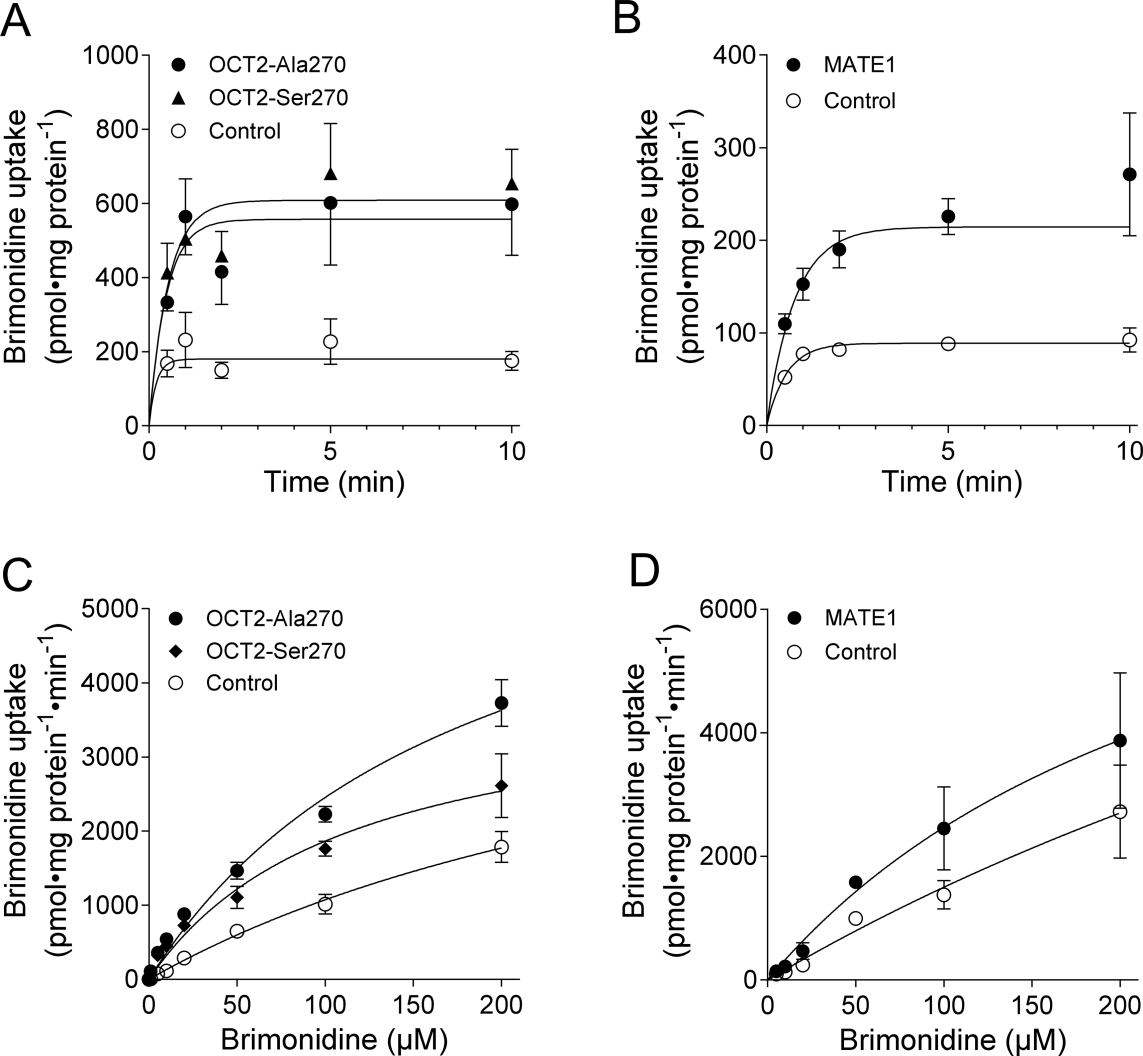
Time and concentration dependence of brimonidine transport by OCT2-Ala270, OCT2-Ser270, and MATE1. (**A**, **B**) For time dependence analysis, OCT2-Ala270–expressing (*filled circles*), OCT2-Ser270–expressing (*filled triangles*), and MATE1-expressing cells (*filled circles*) and vector-transfected control cells (*open circles*) were incubated with 5 µM brimonidine, and cellular uptake was terminated after the indicated time points. (**C**, **D**) Concentration dependence of brimonidine transport by OCT2-Ala270–expressing (*filled circles*), OCT2-Ser270–expressing (*filled diamonds*), and MATE1-expressing cells (*filled circles*) and vector-transfected control cells (*open circles*) was measured after a 30-second incubation time with brimonidine concentrations between 0.1 and 200 µM. Data are mean ± SEM of three determinations, each performed in triplicate.

Transport data were analyzed with GraphPad Prism 10.4.1 (GraphPad Software, La Jolla, CA, USA). The screening experiment (Fig. 1B) was performed in one experiment in triplicate. All other experiments were performed in triplicate on 3 different days and are considered independent experiments (*N* = 3). The means of the triplicates were calculated for each day. Data in Figures 1C–F and 2 are the mean values of these triplicate means and are given with their standard error (SEM).

Nonlinear regression (least squares fit with outlier removal with default Q = 1%) analysis was used for Figures 1E, 1F and 2. The brimonidine concentrations that achieved half-maximum inhibition (IC_50_) of TEA accumulation were determined by fitting the values to the following four-parameter equation^23^: uptake (S) = uptake (min) + (uptake (min) – uptake (max))/(1 + (([S]/IC_50_)^slope^)), where uptake (S) is the TEA uptake at a given brimonidine concentration [S]; uptake (min) and uptake (max) are the minimal and maximal TEA uptake values, respectively; and slope is the Hill slope. Time-dependent uptake values of brimonidine were fitted using the following formula^23^: c_in_ = k_in_/k_out_ * c_out_ * [1 – exp(–k_out_ * t)], where k_in_ and k_out_ are the rate constants for inwardly and outwardly directed transport, respectively; c_in_ and c_out_ are the intracellular and extracellular substrate concentrations, respectively; and t is the time. For the determination of the kinetic parameters K_m_ and V_max_, brimonidine uptake values were first corrected for uptake into vector-transfected control cells. Data were then fitted using the Michaelis–Menten equation^23^: V = (V_max_ * [S])/(K_m_ + [S]), where V is the initial uptake velocity, and [S] is the substrate concentration.^23^ Variations in the K_m_ and V_max_ values are given as asymmetrical profile-likelihood confidence intervals (95% confidence level) and variations in V_max_/K_m_ as the SEM.

To determine statistical significance between control and transporter-transfected cells in the accumulation experiments of Figures 1C and 1D, unpaired *t*-tests were performed. To determine whether time-dependent accumulation (Figs. 2A, 2B) or concentration-dependent accumulation (Figs. 2C, 2D) were significantly higher in the transporter-transfected cells than in control cells, curves were compared using an extra sum-of-squares *F* test. *P* values <0.05 were considered significant.

### Ocular Tissue Acquisition and Preparation

Whole human eyes were enucleated from two patients with glaucomatous eyes and one patient with a nonglaucomatous eye. Eye globes were embedded in paraffin by the University Hospital Tübingen’s Center for Ophthalmology. The study was conducted according to the Declaration of Helsinki and was approved by the Ethics Committee of the University Hospital of Tübingen, Medical Faculty of the Eberhard-Karls-University, Tübingen (approval number 528/2025BO2). Basic patients’ characteristics are given in Table 1.

**Table 1.** Basic Demographic Data of the Patients

Patient	Sex	Age, Y	Diagnosis
1	Male	79	POAG
2	Female	73	POAG
3	Male	66	Uveal melanoma, not glaucomatous

### Immunohistochemistry

For the immunohistochemical staining experiments, the formalin-fixed, paraffin-embedded eye globes were cut into 3-µm-thick sections and transferred to glass slides. Neoclear (Merck Millipore, Darmstadt, Germany) solution was used for deparaffinization, followed by rehydratization with isopropanol (Carl Roth GmbH & Co. KG, Karlsruhe, Germany) and ethanol (Merck Millipore). The samples were incubated in the target retrieval solution buffer at pH 6.0 (Agilent cat. S236984-2) for 30 minutes in a steamer. Subsequently, samples were washed two to three times in TBST buffer (24.2*g* Tris, 84.8*g* NaCl, 2000 mL H_2_O, pH 7.6 + 0.1% Tween). Dako REAL Peroxidation Blocking Solution (Agilent cat. S202386-2) was used to block endogenous peroxidase (5 minutes). Following a washing step, the primary antibody was diluted in Dako REAL antibody dilution buffer (Agilent cat. S202230-2) as follows: (1) anti-OCT2: KEK antibody (rabbit) 1:200 as described in Nies et al.^27^ or (2) anti-MATE1: HPA021987 (rabbit) (Sigma-Aldrich, RRID:AB_1857185) 1:50 as described in Nies et al.,^28^ and samples were incubated overnight at 4°C. The primary antibody was omitted for the negative control and replaced with a normal rabbit immunoglobulin fraction (negative isotype control; Agilent cat. X090302, RRID:AB_906174). For the staining, a dextran-coated peroxidase-coupled polymer system (Dako REAL, EnVision, Detection Kit, Peroxidase/DAB+, Rabbit/Mouse; Agilent cat. K5007, RRID:AB_2888627) was used. Pictures were taken with a slide scanner (Olympus VS120; Olympus, Tokyo, Japan).

### Molecular Structure of Brimonidine

The canonical simplified molecular-input line-entry system string of brimonidine (PubChem CID 2435) was downloaded from PubChem (https://pubchem.ncbi.nlm.nih.gov) and imported into MarvinSketch version 24.3.0 (https://www.chemaxon.com). The structure was displayed using the major microspecies plugin and a pH value of 7.4.

## Results

### Identification of Brimonidine as a Substrate of Organic Cation Transporters

We initially determined whether brimonidine is a substrate of the organic cation transporters OCT1, OCT2, OCT3, MATE1, and MATE2K (Fig. 1B). After an incubation period of 10 minutes, brimonidine accumulated to a greater extent only in OCT2- and MATE1-expressing cells compared with vector-transfected control cells. As accumulation was at least twofold higher than in control cells,^29^ brimonidine was considered a substrate of OCT2 and MATE1. These findings were confirmed by experiments with the organic cation transporter inhibitor MPP.^17^ While brimonidine uptake into HEK cells overexpressing OCT2 (Fig. 1C) and MATE1 (Fig. 1D) was significantly higher in the absence of MPP, the presence of MPP led to a strong reduction of brimonidine uptake. OCT2 and MATE1 were therefore both selected for in-depth functional characterization.

### Characterization of Brimonidine Transport by OCT2 and MATE1

The inhibitory effect of brimonidine on the uptake of the prototypical substrate TEA by OCT2 and MATE1 was investigated in the presence of brimonidine concentrations ranging from 1 to 200 µM (Figs. 1E, 1F). A concentration-dependent inhibition was observed, and curve fitting yielded IC_50_ values for brimonidine of 15.7 µM for OCT2 and 27.7 µM for MATE1.

Next, brimonidine accumulation by transporter-expressing cells was investigated in a time course assay (Figs. 2A, 2B). We also investigated brimonidine transport by OCT2-Ala270Ser, which is a common genetic variant previously associated with altered drug transport function (e.g., of metformin).^19^^,^^30^ Brimonidine was rapidly taken up into transporter-overexpressing HEK cells. The curves between control cells and the respective transporter-expressing cells were significantly different, with *F* and *P* values of *F* = 19.12, *P* < 0.0001 for OCT2-Ala270 versus control (Fig. 2A); *F* = 24.58, *P* < 0.0001 for OCT2-Ser270 versus control (Fig. 2A); and *F* = 85.62, *P* < 0.0001 for MATE1 versus control (Fig. 2B). Since the transport was almost saturated after 1 minute, an incubation time of 30 seconds was chosen for the determination of the kinetic parameters in order to be in the phase of linear uptake.

Kinetic parameters of brimonidine transport were determined using assays in which the brimonidine concentrations ranged from 0.1 to 200 µM at a fixed time point within the linear uptake range (Figs. 2C, 2D). Brimonidine accumulated in a concentration-dependent manner into OCT2-Ala270–, OCT2-Ser270–, and MATE1-expressing cells and exhibited moderate affinity to all three transporters. The curves between control cells and the respective transporter-expressing cells were significantly different, with *F* and *P* values of *F* = 122.5, *P* < 0.0001 for OCT2-Ala270 versus control (Fig. 2C); *F* = 27.67, *P* < 0.0001 for OCT2-Ser270 versus control (Fig. 2C); and *F* = 4.81, *P* = 0.0149 for MATE1 versus control (Fig. 2D). The determined K_m_ values were 17.9 µM for OCT2-Ser270, 37.9 µM for MATE1, and 99 µM for OCT2-Ala270 (Table 2). The maximal transport efficiency V_max_ was approximately threefold higher for OCT2-Ala270 than for OCT2-Ser270. As a measure of transport efficiency, the in vitro intrinsic clearances (V_max_/K_m_) were determined (Table 2). They were in the same range for MATE1 and OCT2-Ala270, and were approximately 1.7-fold higher for OCT2-Ser270 than for OCT2-Ala270.

**Table 2.** Kinetic Parameters of Brimonidine Transport by OCT2-Ala270, OCT2-Ser270, and MATE1

Transporter	K_m_ (95% CI) (µM)	V_max_ (95% CI) (pmol·mg protein^−1^·min^−1^)	V_max_/K_m_ (SEM) (µL·min^−1^·mg protein^−1^)
OCT2-Ala270	99.0 (53–203)	2740 (2102–3987)	27.7 (4.1)
OCT2-Ser270	17.9 (4.8–66)	834 (603–1242)	46.6 (19.1)
MATE1	37.9 (14–110)	793 (539–1242)	20.9 (8.0)

### Immunolocalization of OCT2 and MATE1 in Eyes

**Figure 3.**
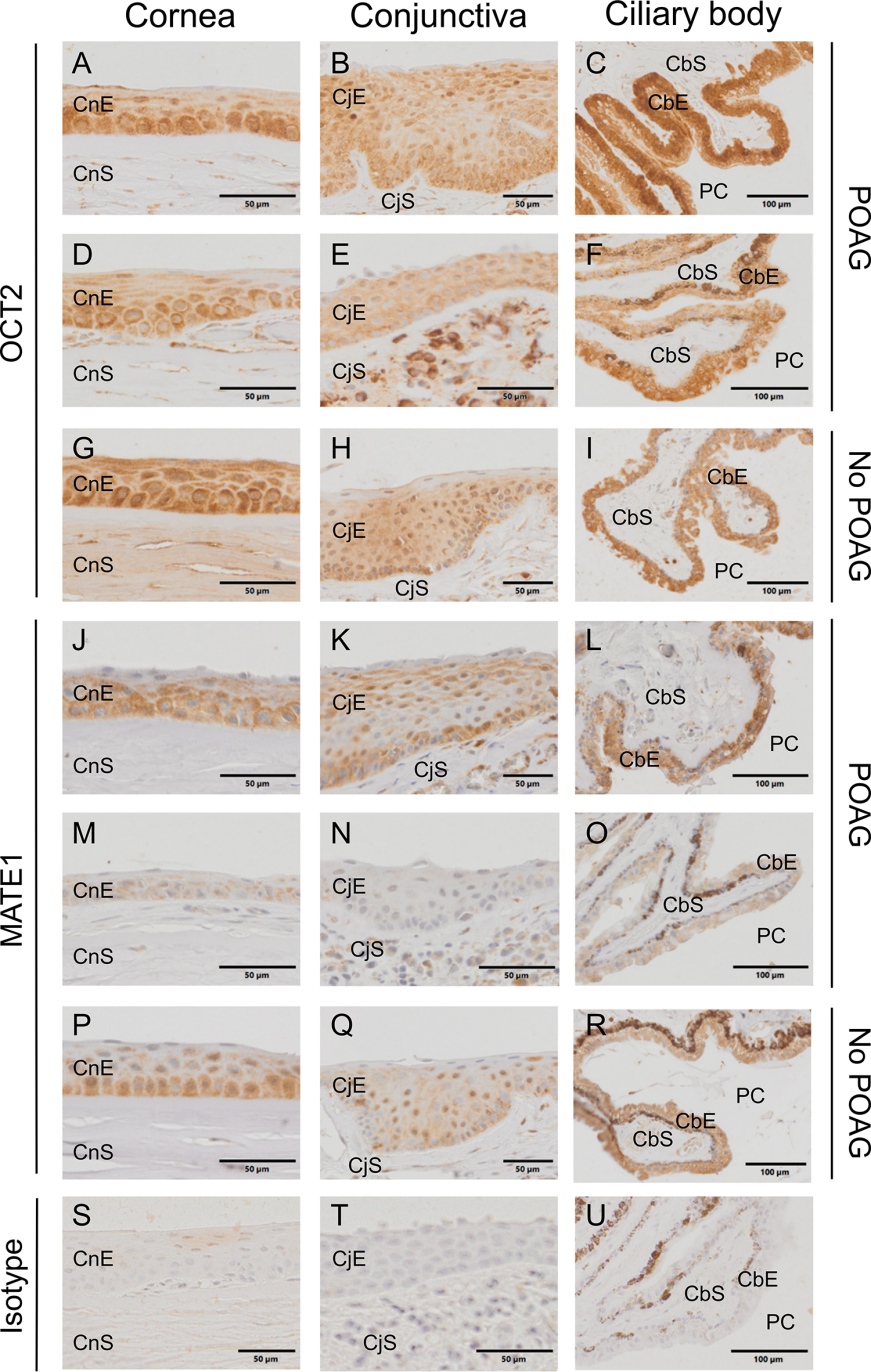
Immunostaining for OCT2 (**A–I**) and MATE1 (**J–R**) in cornea, conjunctiva, and ciliary body in formalin-fixed, paraffin-embedded sections of eyes from two patients with POAG (patient 1: **A–C**, **J–L**; patient 2: **D–F**, **M–O**) and patient 3 with uveal melanoma (**G–I**, **P–R**), using OCT2- and MATE1-specific antibodies diluted 1:200 and 1:50, respectively.^27^^,^^28^ In the isotype negative control stainings (**S–U**), the respective primary antibody was omitted and replaced with a rabbit isotype control antibody. Sections were incubated overnight at 4°C and then stained with a dextran-coated peroxidase-coupled polymer system. Images were taken with a slide scanner. CbE, ciliary body epithelium; CbS, ciliary body stroma; CjE, conjunctival epithelium; CjS, conjunctival stroma; CnE, corneal epithelium; CnS, corneal stroma; PC, posterior chamber.

In order to evaluate the cellular localization of the transporters OCT2 and MATE1 in glaucomatous and nonglaucomatous eyes, immunohistochemical staining experiments were performed and transporter localization was analyzed in the cornea, the conjunctiva, and the ciliary body (Fig. 3). In comparison to the respective isotype control stainings (Figs. 3S–U), both transporters were determined in all tested ocular tissue sections in glaucomatous and nonglaucomatous eyes (Figs. 3A–R). In the cornea, OCT2 staining appeared to be similar in all epithelial cell layers (Figs. 3A, 3D, 3G) of glaucomatous and nonglaucomatous eyes. However, for MATE1, staining was less strong than for OCT2 and more prominent in the epithelial cell layer neighboring the stroma in both glaucomatous and nonglaucomatous eyes (Figs. 3J, 3M, 3P). The localization of OCT2 and MATE1 was also clearly visible in the conjunctiva (Figs. 3B, 3E, 3H, 3K, 3N, 3Q) and in the nonpigmented ciliary epithelium of the ciliary body (Figs. 3C, 3F, 3I, 3L, 3O, 3R) of glaucomatous and nonglaucomatous eyes. Based on the limited approach staining, intensity of OCT2 was comparable between glaucomatous and nonglaucomatous tissues. For MATE1, similar staining intensity was observed in the nonglaucomatous sample and one glaucomatous sample. However, as demonstrated by the glaucomatous tissue samples, the intensity of staining can differ for one disease entity (Fig. 3).

## Discussion

Ocular transporters may play a critical role in the disposition of ocular drugs, yet little is currently known about their expression, function, and substrate spectra in human glaucomatous eyes. Brimonidine, a frequently prescribed drug, must pass through the cornea and the conjunctiva after topical administration to reach its target site in the ciliary body, where it influences aqueous humor production.^9^ Previous studies have suggested that an active transport process may contribute to brimonidine uptake in ocular tissues.^31^^,^^32^ Because brimonidine is a cationic drug, the involvement of an organic cation transport process in brimonidine transport has been hypothesized,^31^ but the molecular identity of the transporters involved remains unknown.

In this study, we therefore systematically investigated the role of the organic cation transporters OCT1, OCT2, OCT3, MATE1, and MATE2K in brimonidine transport using transporter-transfected cells. These transporters are well known for their ability to transport a wide variety of cationic compounds, including a considerable number of clinically relevant drugs (reviewed, e.g., in various studies^16^^,^^17^^,^^19^). Of the tested SLC transporters (OCT1, OCT2, OCT3, MATE1, MATE2K), significant uptake of brimonidine was observed only in HEK cells overexpressing OCT2 and MATE1. The uptake levels were more than twofold higher than in control cells, thereby meeting the established threshold for substrate classification.^29^ An approach using small interfering RNA (siRNA) knockdown of OCT2 or MATE1 in current human corneal epithelial cell line models was not possible because data indicate that OCT2 and MATE1 expression is not present or negligible in primary human corneal epithelial cells or SV40-immortalized corneal epithelial cells,^33^ respectively. We therefore focused in this study on robust and well-controlled heterologous HEK expression systems to establish mechanistic evidence for OCT2- and MATE1-mediated brimonidine transport.

OCTs and MATEs are often regarded as “polyspecific” organic cation transporters with overlapping substrate specificities.^17^^,^^34^^–^^37^ Nevertheless, each transporter apparently has a unique substrate profile and selectivity, as supported by the lack of brimonidine transport by OCT1, OCT3, and MATE2K. Our findings are corroborated by the identification of other compounds that are selectively transported by specific OCTs and MATEs (overview in Koepsell^17^). For instance, the anticholinergic agent homatropine is transported by OCT2 and OCT3 but not by OCT1, MATE1, and MATE2K.^38^ Another example is methamphetamine, which was identified as a substrate of OCT2, MATE1, and MATE2K but not of OCT1 and OCT3.^39^ Computational methods such as ligand- and structure-based molecular modeling (reviewed, e.g., in Türková et al.^40^) and machine learning approaches^36^ have advanced the understanding of the substrate selectivity of different transporters. However, it is currently not possible to predict whether a compound will actually be transported. It can be hypothesized that docking studies using three-dimensional structural models, which are now available from AlphaFold^41^ or derived from cryo-EM analyses,^42^^,^^43^ will lead to more accurate substrate predictions in the future.

We demonstrated saturation of OCT2- and MATE1-mediated brimonidine uptake after about 1 minute. This rapid uptake aligns with the short residence time of topical eye drops on the cornea of only a few minutes,^15^ supporting the physiological relevance of transporter-mediated uptake during the brief window of drug availability. We also demonstrated that OCT2 and MATE1 transport brimonidine with moderate affinity, as indicated by K_m_ values of 99 and 37.9 µM, respectively. Application of the prototypical organic cation transport inhibitor MPP^+^^17^ further confirmed the transporter-mediated nature of brimonidine accumulation, as MPP^+^ markedly reduced intracellular brimonidine concentrations in both OCT2- and MATE1-overexpressing cells. The interaction of brimonidine with OCT2 and MATE1 was also corroborated by the inhibitory effect of brimonidine on the OCT2- and MATE1-mediated uptake of TEA, a prototypical OCT and MATE substrate. The IC_50_ values of 15.7 µM (for OCT2) and 27.7 µM (for MATE1) fall within the previously reported range of IC_50_ values for other drugs that interact with OCT2 and MATE1.^17^^,^^35^ Ueda et al.^31^ showed that an organic cation transport process is involved in brimonidine transport because brimonidine inhibits the uptake of guanidine, a prototypical substrate of organic cation transporters, in isolated rabbit conjunctival epithelium. Since guanidine uptake was also inhibited by TEA and choline, the authors concluded from this inhibitor profile that organic cation transport was not mediated by OCT3, OCTN2, or the OC/H^+^ antiporter. However, the relevant organic cation transporters were not identified at the molecular level.^31^ Since OCT2 is inhibited by choline and TEA^44^ and MATE1 by TEA,^45^ the inhibitor profile reported by Ueda et al.^31^ is consistent with OCT2 and MATE1 being the relevant transporters for the uptake of brimonidine in the conjunctiva. Different brimonidine uptake systems appear to exist in human retinal ARPE-19 cell monolayers, where brimonidine uptake was not inhibited by MPP^+^.^32^

We also investigated the impact of genetic variation. Unlike the genetically polymorphic OCT1, there is only one common genetic variant in OCT2 (reviewed in two studies^16^^,^^19^). While reduced transport by OCT2-Ser270 has been described for certain drugs, such as metformin,^19^^,^^46^ other studies have shown increased metformin transport^30^ or unchanged trimethylamine-*N*-oxide transport.^47^ We found that OCT2-Ser270 exhibited a lower K_m_ value (17.9 µM) and, by implication, a significantly higher affinity for brimonidine compared to wild-type OCT2-Ala270. This enhanced affinity, combined with a lower V_max_ value, resulted in a 1.7-fold increase in transport efficiency for the variant. These results support the notion of a substrate-specific dependency of transport similar to OCT1, underlining the complexity of ligand binding to OCT2.^48^ Moreover, our results suggest that genetic variability in OCT2 may influence brimonidine pharmacokinetics, a hypothesis that warrants investigation in patient cohorts in future studies.

**Figure 4.**
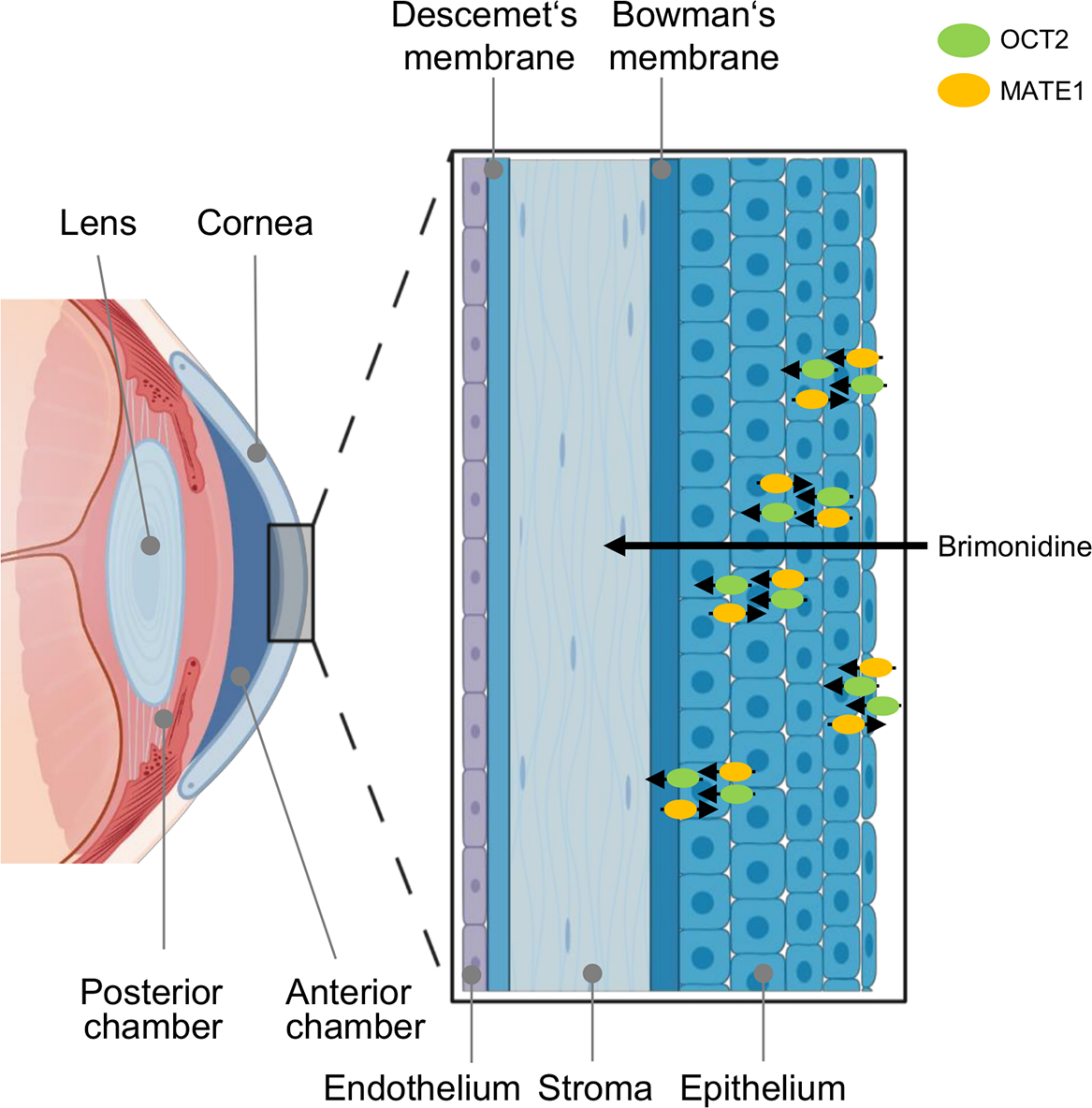
Schematic diagram showing the proposed brimonidine pathway following the corneal route from the cornea into the ciliary body. After topical application, brimonidine is transported through the cornea into the anterior chamber and then diffuses through the pupil into the posterior chamber. From there, it is transported into the ciliary body, where the target α_2_-receptors reside. OCT2 and MATE1 contribute to brimonidine uptake at the apical membrane, promoting intracellular accumulation within corneal epithelial cells. Subsequently, brimonidine will cross the basolateral membrane via OCT2, as an electrogenic facilitative diffusion process that translocates organic cations in both directions along with their concentration gradients. MATE1 present at the basolateral membrane has the capacity to counteract efflux through brimonidine reuptake. The overall transcellular flux across the corneal epithelium is dependent on the balance between these competing transport processes. The diagram was partially created with BioRender.

The functional importance of our findings regarding brimonidine uptake through OCT2 and MATE1 is supported by our immunohistochemistry data, which revealed OCT2 and MATE1 protein expression in the cornea, the conjunctiva, and the nonpigmented epithelium of the ciliary body. Figure 4 shows a diagram of the proposed pathway of brimonidine transport. According to the corneal route, which is the main route of topical ocular drug delivery,^49^ brimonidine is transported across the corneal epithelium involving OCT2 and MATE1, reaching the aqueous humor in the anterior chamber. It then diffuses across the pupil into the posterior chamber, from which it is absorbed by the ciliary body. The expression of both transporters in the ciliary body is particularly relevant, as it plays a central role in aqueous humor production and is the primary target of antiglaucoma agents.^9^ The expression patterns further suggest that both OCT2 and MATE1 may mediate the pharmacodynamic effects of brimonidine in the anterior and posterior sections of the eye by influencing its local availability.

Given the established clinical efficacy of brimonidine, its net flux must be directed across the corneal epithelium into the anterior chamber, as shown in Figure 4. This would be consistent with a transcellular route, in which brimonidine traverses successive layers of corneal epithelial cells via alternating uptake and efflux steps. It appears that OCT2 and MATE1 are expressed in the apical and basolateral membranes of corneal epithelial cells, although current immunohistochemical approaches do not allow definitive resolution of membrane-specific expression. We hypothesize that OCT2 and MATE1 contribute to brimonidine uptake at the apical membrane, facilitating intracellular accumulation within corneal epithelial cells. Subsequent movement across the basolateral membrane could then occur via OCT2, which is an electrogenic facilitative diffusion system that translocates organic cations in both directions along with their concentration gradients.^35^ In this context, MATE1 present at the basolateral membrane could potentially oppose this efflux by mediating brimonidine reuptake, thereby modulating the overall rate and directionality of transcellular transport. The net flux across the corneal epithelium would thus reflect the balance of these partially competing transport processes.

The expression of OCT2 and MATE1 might be altered by chronic glaucoma or long-term use of topical medications. Regulation of transporter expression is a complex process occurring on several different levels. Most mechanistic insights to date are derived from cell lines or rodent models, and it is not known whether these results translate to humans.^50^ For OCT2, we have previously demonstrated substantial interindividual variability in tissue samples from patients with clear-cell renal carcinoma due to alterations in DNA methylation.^51^ In contrast, comparable data for MATE1 in clinical human samples are currently lacking.^50^ These findings indicate that transporter expression can, in principle, be modulated under specific pathological conditions. However, there is currently no evidence that chronic glaucoma and the long-term use of topical medications alter OCT2 or MATE1 expression in the human eye. This topic will be an interesting area of future research.

A limitation of this study is the small number of human ocular specimens analyzed by immunohistochemistry, which may limit generalizability. The exclusive use of diseased tissue reflects practical and ethical constraints, as access to suitable healthy donor tissue is very limited, and postmortem tissue is not feasible for valid transporter staining. Importantly, there is no evidence to suggest that OCT2 and MATE1 expression is inherently linked to the underlying disease because expression patterns were consistent across the analyzed specimens. Larger future studies with human ocular specimens will be necessary to confirm the observed expression patterns. Also, future in vivo or animal studies would be an important next step to further underline the role of OCT2 and MATE1 in brimonidine transport.

## Conclusions

Our data demonstrate the involvement of OCT2 and MATE1 in the transport of brimonidine in anterior eye sections. Both transporters are expressed at the protein level in these structures and can mediate brimonidine uptake. Consequently, both transporters may influence the pharmacodynamic effects of brimonidine. Variants of the transport proteins may significantly alter transport efficiency and thereby pharmacokinetics of brimonidine, as shown by the common OCT2 variant Ser270. Collectively, these findings enhance our understanding of brimonidine's transporter-mediated disposition. The knowledge of brimonidine's transporter-mediated disposition could facilitate the development of personalized therapeutic strategies, particularly for individuals with genetic variants that affect transporter function. Future clinical and pharmacogenetic studies are needed to clarify the clinical relevance of these transport processes in patients with glaucoma.
